# A catalase nanocapsule inhibits VSMCs proliferation and migration through PTEN/NLRP3 pathway

**DOI:** 10.3389/fphar.2026.1826703

**Published:** 2026-05-08

**Authors:** Yudan Wang, Yu Chen, Wenjuan Quan, Taoli Sun, Zhe Shi, Qinhui Tuo

**Affiliations:** 1 Key Laboratory for Quality Evaluation of Bulk Herbs of Hunan Province, Hunan University of Chinese Medicine, Changsha, Hunan, China; 2 Key Laboratory for Novel Antibody Drugs and Intelligent Delivery System of Hunan Province,Hunan University of Medicine, Huaihua, China; 3 Key Laboratory of Vascular Biology and Translational Medicine, Hunan University of Chinese Medicine, Changsha, Hunan, China

**Keywords:** catalase nanocapsule, inflammation, phenotype switch, pten, vascular smooth muscle cells

## Abstract

**Background:**

Aberrant VSMC proliferation, migration, and phenotypic switching drive vascular remodeling. Oxidative stress is pivotal, but effective antioxidants are limited.

**Methods:**

A catalase-loaded nanocapsule (CAT-nc) was developed for sustained ROS scavenging in Ang II-induced VSMCs.

**Results:**

CAT-nc reduced ROS, inhibited proliferation/migration, restored contractile phenotype via PTEN reactivation and NLRP3 suppression; PTEN inhibition abolished these effects.

**Discussion:**

Confirming the ROS-PTEN-NLRP3 axis, CAT-nc attenuates oxidative stress-driven VSMC switching, offering a promising nanotherapy for vascular diseases.

## 1 Introduction

Cardiovascular diseases (CVDs) remain a leading cause of morbidity and mortality worldwide, with pathological vascular remodeling representing a central process driving disease progression (Zhao et al., 2019; Lv et al., 2024). A key cellularevent underlying vascular remodeling is the phenotypic switching of vascular smooth muscle cells (VSMCs) from a contractile phenotype to a synthetic, proliferative, and migratory state (Qi et al., 2019; Muñoz et al., 2025; Siracusa et al., 2025). This phenotypic transition directly contributes to neointimal hyperplasia, atherosclerosis, and restenosis, making VSMC plasticity an important therapeutic target (Muñoz et al., 2025). Excessive production of reactive oxygen species (ROS) not only induces cellular damage but also functions as a critical signaling mechanism that activates redox-sensitive pathways involved in proliferation, migration, and inflammation (Förstermann et al., 2017; Incalza et al., 2018). Angiotensin II (Ang II), a major effector of the renin–angiotensin system, robustly stimulates ROS generation in VSMCs and promotes pathological phenotypic remodeling (Shi et al., 2020; Shaito et al., 2022). Accordingly, antioxidant strategies aimed at restoring redox homeostasis have emerged as promising approaches for mitigating vascular dysfunction.

Catalase (CAT) is a key endogenous antioxidant enzyme that catalyzes the decomposition of hydrogen peroxide and maintains intracellular redox balance. Despite its potent ROS-scavenging capacity, the therapeutic application of native catalase is severely limited by poor stability, rapid degradation in biological fluids, and insufficient tissue distribution (Baker et al., 2023; Shi et al., 2020; D’Agostino et al., 2025). These intrinsic limitations have markedly restricted the translational potential of catalase-based antioxidant therapy for cardiovascular diseases (Radomska-Leśniewska et al., 2017; Jomova et al., 2024). Recent advances in nanotechnology have provided effective strategies to overcome these challenges. Nano-encapsulation can protect enzymatic activity, enhance structural stability, and improve cellular uptake of therapeutic proteins (Soumya and Raghu, 2023; Choudhury et al., 2024). In particular, nanocapsule systems that individually encapsulate enzyme molecules within a biocompatible polymer shell have demonstrated sustained catalytic activity and improved biological performance (Khan et al., 2025; Li et al., 2025). However, whether nano-encapsulated catalase can effectively regulate VSMC phenotypic switching and the underlying molecular mechanisms remain largely unexplored.

At the molecular level, phosphatase and tensin homolog (PTEN) is a redox-sensitive regulator of cellular homeostasis whose activity can be impaired under oxidative stress conditions (Nguyen Huu et al., 2021). Loss of PTEN function promotes downstream inflammatory signaling, including activation of the NLRP3 inflammasome, which has been implicated in vascular inflammation and VSMC dysfunction (Chow and Salmena, 2020; Huang et al., 2020). Although both PTEN and NLRP3 are known to participate in oxidative stress-related vascular pathology, whether antioxidant enzyme–based nanotherapeutics can modulate this signaling axis to regulate VSMC behavior has not been clearly defined.

In this study, we developed a catalase nanocapsule (CAT-nc) delivery system and investigated its effects on Ang II–induced VSMC proliferation, migration, and phenotypic switching. By integrating network pharmacology analysis with systematic *in vitro* experiments, we demonstrate that CAT-nc effectively scavenges intracellular ROS, restores PTEN activity, suppresses NLRP3 inflammasome signaling, and thereby inhibits pathological VSMC dysfunction. These findings provide mechanistic insight into antioxidant nanotherapy-mediated regulation of vascular remodeling and highlight CAT-nc as a potential therapeutic strategy for oxidative stress–associated vascular diseases.

## 2 Materials and methods

### 2.1 Network pharmacological analysis

#### 2.1.1 Prediction of targets for CAT in treating cardiovascular diseases

Potential targets of catalase (CAT) were retrieved from the GeneCards database (https://www.genecards.org/) using “CAT” as the search keyword (Stelzer et al., 2016). Targets associated with cardiovascular diseases were obtained by searching the same database with the keyword “vascular remodeling”The intersecting targets between CAT-related proteins and vascular remodeling related targets were identified using Venny 2.1.0 and considered potential targets for subsequent exploratory analysis.

#### 2.1.2 Cardiovascular diseases construction of the protein-protein interaction (PPI) network

The intersecting targets were imported into the STRING database (https://string-db.org/) to construct a PPI network (Szklarczyk et al., 2023). The species was restricted to *Homo sapiens*, and the minimum required interaction score was set to 0.400. The resulting network data were imported into Cytoscape software (version 3.9.0). Network topological analysis was performed using the CytoNCA plugin, and nodes were ranked according to degree values to identify core targets within the network.

#### 2.1.3 NetworkGO and KEGG pathway enrichment analysis of core target proteins for CAT in treating cardiovascular diseases

The DAVID Bioinformatics Resources 6.8 online analysis platform (DAVID Functional Annotation Bioinformatics Microarray Analysis (ncifcrf.gov) was utilized to perform GO and KEGG enrichment analyses on the core targets of CAT for cardiovascular diseases (Sherman et al., 2022). The top five enriched BP and MF terms and the top ten KEGG pathways were selected for visualization using bubble charts.

#### 2.1.4 Molecular docking verification

The CAT, PTEN, and NLRP3 were selected for molecular docking. The corresponding three-dimensional structures of the proteins were downloaded as PDB files from the RCSB Protein Data Bank (https://www.rcsb.org/). Protein-protein interaction docking analyses were then performed using the HDOCK online webserver, and the resulting docking models were visualized with PyMol version 2.4.1.

### 2.2 CAT-nc characterization and drugs

#### 2.2.1 Characterization and stability of CAT-nc

Catalase nanocapsule was prepared following the previously reported method (Qin et al., 2020). The average particle size of CAT-nc is approximately 25 nm, with a distribution range of 20–30 nm, and its zeta potential is +1.5 mV. After incubation in PBS at 37 °C for 24 h, CAT-nc retains 90% of its initial enzymatic activity. Following co-incubation with 50 μg/mL trypsin at 37 °C for 2 h, CAT-nc retains 87% of its activity. Moreover, after storage at 4 °C and 25 °Cfor 3 months, CAT-nc still retains 100% of its activity. Furthermore, after freeze-drying treatment, CAT-nc retains over 90% of its activity.

#### 2.2.2 Drugs

The outer layer of CAT-nc used 2-methacryloyloxyethyl phosphorylcholine (MPC), N-(3-aminopropyl) methacrylamide hydrochloride (APM), and N,N′-methylenebisacrylamide (BIS) to form a polymeric thin shell. The powder was dissolved in 1x PBS and stored at 4 °C. Angiotensin-II (Ang II, A107852) was purchased from Aladdin Biochemical Technology (Shanghai, China). ROS scavenger N-acetylcysteine (NAC, A9165) was purchased from Sigma-Aldrich (St. Louis, United States); PTEN inhibitor VO-Ophic (S8174) was purchased from Selleck Chemicals (Houston, United States). N-acetylcysteine (NAC, A9165, Sigma-Aldrich, St. Louis, United States) was used at a final concentration of 5 mM in the co-treatment experiments.The Mouse IL-1 beta ELISA Kit (KE10003) was purchased from Proteintech.

### 2.3 Cell culture and treatment

The MOVAS cell line (mouse aortic vascular smooth muscle cells, catalog number STM-CL-6503) was purchased from Xiangya Central Laboratory (Changsha, China) and used within passage 10 (≤P10) for all experiments. High-glucose DMEM medium (PM150210), PBS buffer (PB180327), and 0.25% trypsin (PB180224) were purchased from Wuhan Ponosay Life Technology (Wuhan, China). Fetal bovine serum (13011-8611) was purchased from Zhejiang Tianhang Biotechnology (Hangzhou, China). Cells were incubated with high-glucose DMEM medium supplemented with 10% fetal bovine serum, 100 U/ml penicillin and 100 μg/ml streptomycin at 37 °C in a humidified incubator with 5% CO^2^.

### 2.4 Cell viability assay

Cells were seeded in 96-well plates (1 × 10^4^ per well). To determine the dosage-dependent effects on cell viability, cells were pretreated with CAT-nc at the dosage of 0.1, 0.3, 1, 3, 10 μg/mL for 2 h and then co-incubated with Ang II (1 μM) for another 24 h. On the second day CCK-8 test was conducted. To determine the time-dependent effects on cell viability, cells were pretreated with CAT-nc at the dosage of 0.3 μg/mL for 2 h and then co-incubated with Ang II (1 μM) for another 24, 48 and 72 h. Then, CCK-8 test was conducted. CCK-8 kit (BS350B) was purchased from Guangzhou Biosharp Biotechnology (Guangzhou, China). After 100ul of CCK-8 reagent diluted 1:10 times was added to each well, cells were incubated was at 37 °C for 1 h. Then, optical density (OD) was detected at a wavelength of 450 nm using a microplate reader.

### 2.5 DCFH-DA assay

ROS fluorescent probe DCFH-DA kit (E-BC-K138F) were purchased from Wuhan Ilerite Biotechnology (Wuhan, China). Cells in logarithmic phase were seeded in 6-well plates at a density of 1 × 10^6^ (cells/well). After 24 h incubation, culture medium was removed. Reagent 3 was prepared as instructed and then added to each well. After rinsing for 2 times, 1 mL of DCFH-DA was added. The mixture was incubated at 37 °C for 15 min. After incubation, the reagent was added and washed three times. The maximum excitation wavelength and emission wavelengths were 488/525 nm. Images were taken with a fluorescence microscope.

### 2.6 Wound healing assay

Cells were seeded at a density of 1 × 10^6^ (cells/well) in 6-well plates. A 20 μL tip was used to scratch the monolayer in each well. After scratching the monolayer, cells were washed twice with PBS and then incubated in DMEM containing 0.5% fetal bovine serum (FBS) for 24 h to minimize the contribution of cell proliferation to wound closure. Subsequently, they were observed and photographed under a microscope at 0 h and 24 h.

### 2.7 Flow cytometry

Protease inhibitors (P0100) and PI staining solution (C0080) were purchased from Beijing Solaibao Technology (Beijing, China). Cells were seeded in 10 cm dishes. After treatment, cells were digested with 0.25% trypsin and collected. The suspension was centrifuged at 1000 rpm for 5 min. Each tube was fixed overnight at 4 °C with 75% alcohol. After fixation, tubes were washed 2 times with precooled PBS. Subsequently, PI staining solution was added. The mixture was incubated in a 37 °C water bath for 30 min. The cell cycle distribution was detected by flow cytometry.

### 2.8 Western blotting

Cells were washed three times with pre-cooled PBS. The mixture of RIPA lysate (CW2333s, Beijing Kangwei Century Biotechnology, China) and PMSF was added in each dishes. Cells were lysed on ice for 15 min. The protein was scraped off with a cell scraper. The extracts were centrifuged at 12000 rpm for 15 min at 4 °C. Protein concentration was calculated by BCA protein quantification (70-PQ0012, Hangzhou Lianke Biotechnology, China). Protein extracts were separated by electrophoresis on 8%–12% polyacrylamide gel and transferred onto PVDF membrane. Then, the membrane was incubated with primary antibody overnight at 4 °C. Primary antibodies SM22α (60213-1-Ig), α-SMA (14395-1-AP), AKT (10176-2-AP), P-AKT (66444-1-Ig), NLRP3 (19771-1-AP) and GAPDH (10494-1-AP) were purchased from Wuhan Shawk Biotechnology (Wuhan, China). Primary antibodies PTEN (Ab9188) and Osteopontin (OPN) (Ab8448) were purchased from Abcam (Cambridge, UK). Then, the membrane was washed 3 times by TBST and incubated with secondary antibodies for 2 h at room temperature. Sheep anti-mouse secondary antibody (E-AB-1001), sheep anti-rabbit secondary antibody (E-AB-1003) were purchased from Wuhan Ilerite Biotechnology (Wuhan, China). At last, the membrane was washed 4 times with TBST and visualized by chemiluminescence.

### 2.9 Statistical analysis

All data are presented as mean ± SEM. Statistical analysis was performed using SPSS software (version 23.0). Comparisons between two groups were conducted using an independent samples t-test, and comparisons among multiple groups were performed using one-way analysis of variance (ANOVA) followed by Dunnett’s post-hoc test for comparisons against the control or model group. A *P < 0.05* was considered statistically significant.

## 3 Results

### 3.1 Network pharmacology and molecular docking

To preliminarily explore potential molecular targets and signaling pathways associated with catalase in vascular remodeling, a network pharmacology analysis was performed as a hypothesis-generating approach. Candidate targets of catalase were collected from publicly available databases and intersected with vascular remodeling-related genes. PPI network analysis identified several nodes with relatively high connectivity, among which PTEN and NLRP3 were notable. Functional enrichment analysis further suggested that these candidate targets were mainly involved in oxidative stress regulation and inflammatory signaling pathways. These results provided a theoretical basis for subsequent experimental validation.

**FIGURE 1:**
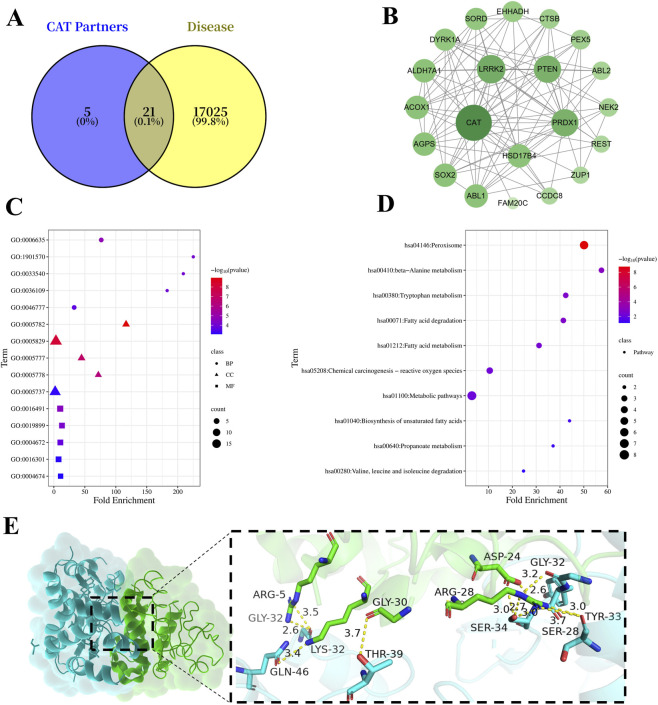
Network Pharmacology and Molecular Docking. **(A)** Intersection of CAT Targets and Cardiovascular Disease Targets. **(B)** PPI Network of Core Target Proteins for CAT in Treating Cardiovascular Diseases. **(C)** Bubble Chart of GO Enrichment Results. **(D)** Bubble Chart of KEGG Pathway Enrichment Results. **(E)** Molecular Docking of CAT and PTEN. Note: Blue represents CAT, green represents PTEN.

Network pharmacology analysis of targets corresponding to CAT revealed 21 intersecting targets (including CAT) between CAT and cardiovascular diseases, as shown in Figure 1A. The interaction information of these 21 potential core target proteins was further imported into Cytoscape 3.9.0 to construct the PPI network for CAT in treating cardiovascular diseases (Figure 1B). The node size and color depth represent the degree value of the targets. As shown in Figure 1C, the top 10 targets ranked by degree value were CAT, LRRK2, PTEN, PRDX1, HSD17B4, ALDH7A1, AGPS, ACOX1, SOX2, and ABL1. The top five cellular component (CC) terms were GO:0005782 (peroxisomal matrix), GO:0005829 (cytosol), GO:0005777 (peroxisome), GO:0005778 (peroxisomal membrane), and GO:0005737 (cytoplasm), indicating that the predicted targets were mainly localized to peroxisome-related structures and cytoplasmic compartments. Peroxisomes are known to participate in lipid metabolism and intracellular redox regulation, suggesting a potential association between the identified targets and oxidative stress related cellular processes (Ding et al., 2021). The top five molecular function (MF) terms included oxidoreductase activity, enzyme binding, and protein kinase related activities. These enriched functions are primarily involved in redox reactions and signal transduction, which are closely associated with cellular responses to oxidative stress (Rhee and Kil, 2017; Han et al., 2024). As shown in Figure 1D, KEGG pathway enrichment analysis identified 11 pathways, among which peroxisome, fatty acid degradation, and fatty acid metabolism were the most significantly enriched. Many of these pathways are involved in metabolic regulation and redox homeostasis (Guan et al., 2019; Ganamurali and Sabarathinam, 2026). Given the well-recognized role of oxidative stress in vascular cell dysfunction, these enriched pathways were considered to offer a theoretical basis for subsequent experimental investigation.

Molecular docking between CAT and PTEN was performed using the HDOCK online server, yielding a binding score of −208.05, which indicates strong binding affinity. The docking conformation is shown in Figure 1E.

### 3.2 CAT-nc significantly inhibited the proliferation of VSMCs

**FIGURE 2:**
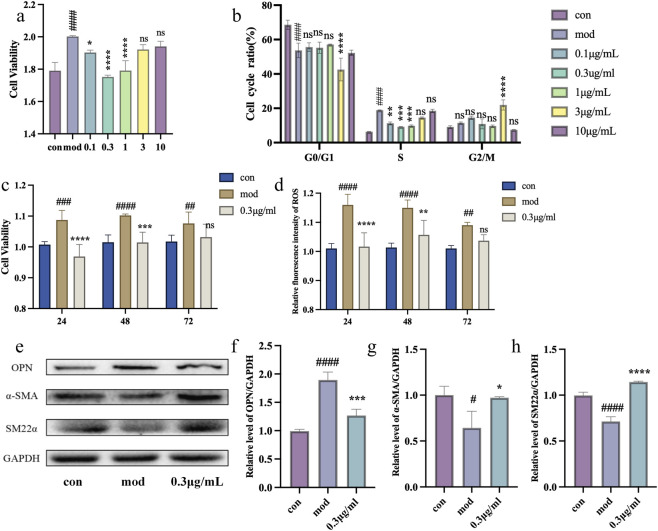
CAT-nc inhibited Ang II-induced proliferation and migration in VSMCs. **(a)** VSMCs proliferation and **(b)** Cell cycle ratio after CAT-nc treatment at different dosage; **(c)** VSMCs proliferation and **(d)** ROS production level after CAT-nc treatment at 0.3 μg/ml at different time; **(e)**–**(h)** Relative protein expressions of SM22α, α-SMA and OPN. Data are expressed as mean ± SE (n = 4 independent biological experiments). ^*^ *P < 0.05,* ^**^ *P < 0.01,* ^***^ *P < 0.001,* ^****^ *P < 0.0001*, compared with model; ^*#*^ *P < 0.05,* ^*##*^ *P < 0.01,* ^*###*^ *P < 0.001,* ^*####*^ *P < 0.0001*, compared with control.

As shown in Figure 2a, angiotensin II (Ang II) treatment significantly promoted the proliferation of VSMCs. CAT-nc effectively inhibited VSMC proliferation at doses of 0.1, 0.3, and 1 μg/ml, while no inhibitory effect was observed at doses of 3 and 10 μg/ml. To further investigate the effect of CAT-nc on the cell cycle, flow cytometry was performed to analyze the proportion of cells in the S phase. The results demonstrated that CAT-nc treatment significantly reduced the number of cells entering the S phase (Figure 2b). This effect was most pronounced at doses of 0.1, 0.3, and 1 μg/ml, with 0.3 μg/ml showing the most significant cell cycle arrest. Based on this finding, 0.3 μg/ml was selected for subsequent time-dependent experiments. As shown in Figure 2c, the inhibitory effect of CAT-nc at this concentration persisted for up to 48 h, indicating its sustained efficacy over an extended period.

In the detection of ROS levels, fluorescence intensity measurements revealed that treatment with 0.3 μg/ml CAT-nc for 48 h markedly decreased intracellular ROS fluorescence intensity (Figure 2d), suggesting that CAT-nc possesses ROS-scavenging ability under these conditions. Furthermore, CAT-nc treatment modulated the expression of key phenotypic markers in VSMCs. As shown in Figures 2e–h, the expression of the synthetic phenotype marker osteopontin (OPN) was significantly downregulated. In contrast, the expression levels of the contractile phenotype markers α-smooth muscle actin (α-SMA) and smooth muscle protein 22-α (SM22α) were notably upregulated.These results suggest that CAT-nc suppresses VSMC proliferation by promoting a phenotypic shift from a synthetic to a contractile state.

### 3.3 CAT-nc regulates VSMCs phenotypic switching by reducing ROS production

**FIGURE 3:**
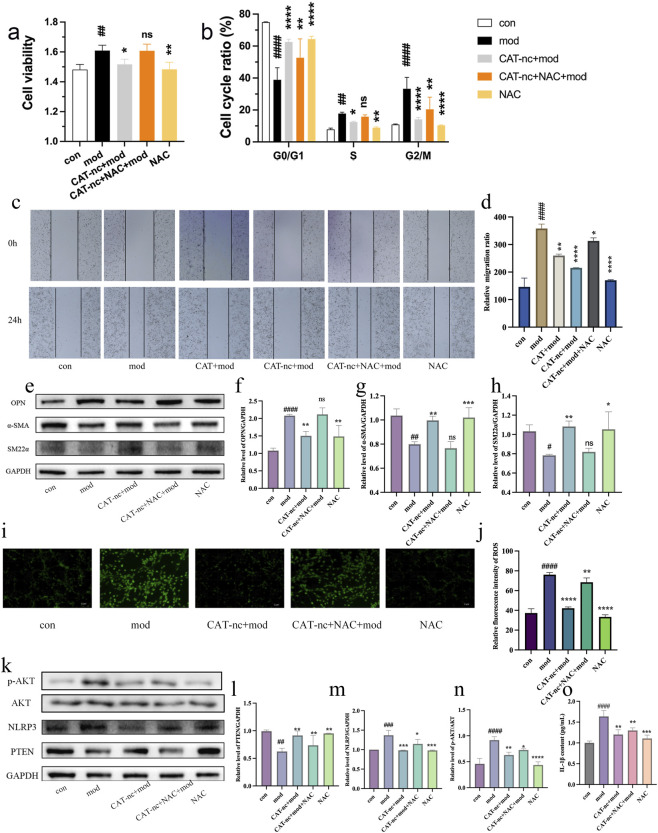
CAT-nc suppressed phenotypic transformation in VSMCs via scavenging ROS production. **(a)** VSMCs proliferation, **(b)** cell cycle ratio and **(c,d)** migration after CAT-nc and NAC treatment correspondingly; **(e)**–**(h)** Relative protein expressions of SM22α, α-SMA and OPN; **(i,j)** ROS production level; **(k)**–**(n)** Relative protein expressions of PTEN, NLRP3, P-AKT and AKT; **(o)** Relative content of IL-1β. Data are expressed as mean ± SE (n = 4 independent biological experiments). ^*^ *P < 0.05,* ^**^ *P < 0.01,* ^***^ *P < 0.001,* ^****^ *P < 0.0001*, compared with model; ^*#*^ *P < 0.05,* ^*##*^ *P < 0.01,* ^*###*^ *P < 0.001,* ^*####*^ *P < 0.0001*, compared with control.

To investigate whether CAT-nc affects the phenotypic switching of VSMCs by inhibiting ROS generation and modulating the PTEN/NLRP3 signaling pathway, we conducted further mechanistic studies. The ROS scavenger N-acetylcysteine (NAC) was used as a positive control in the experiments. The results showed that CAT-nc treatment significantly inhibited Ang II-induced VSMCs proliferation and migration (Figures 3a–d). Regarding the expression of phenotypic marker proteins, compared with the model group, CAT-nc intervention markedly downregulated the expression of the synthetic phenotype marker OPN, while significantly upregulating the expression of the contractile markers α-SMA and smooth muscle protein SM22α (Figures 3e–h). These findings indicate that CAT-nc promotes the transition of VSMCs from a synthetic to a contractile phenotype.

At the level of oxidative stress, ROS fluorescence detection revealed that CAT-nc effectively reduced intracellular ROS fluorescence intensity (Figures 3i,j). Concurrently, analysis of related signaling pathway proteins demonstrated that CAT-nc significantly increased PTEN protein expression and decreased the expression level of the NLRP3 inflammasome (Figures 3k–m). CAT-nc significantly reduced the expression level of the AKT protein and decreased the inflammatory expression level of IL-1β (Figures 3n,o).

Notably, treatment with NAC alone produced phenotypic regulatory effects like those in the normal control group. However, when CAT-nc was administered in combination with NAC, the effects of CAT-nc on inhibiting VSMCs proliferation and migration, as well as on regulating phenotypic marker proteins, were almost completely reversed. This result suggests that the regulatory role of CAT-nc in VSMCs phenotypic switching may depend on its ability to reduce intracellular ROS levels, and that ROS scavenging could be an upstream key event in CAT-nc’s mechanism of action. Taken together, these findings collectively suggest that CAT-nc may inhibit pathological phenotypic switching in VSMCs by reducing ROS generation and subsequently modulating the PTEN/NLRP3 signaling pathway.

### 3.4 CAT-nc regulates the phenotypic switching of VSMCs by increasing the expression of PTEN

**FIGURE 4:**
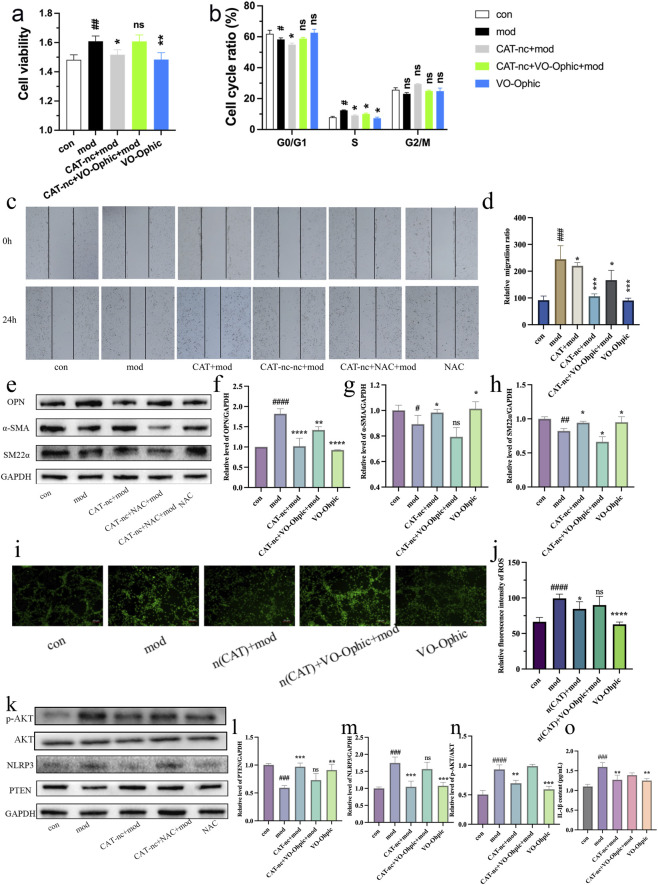
CAT-nc suppressed phenotypic transformation in VSMCs via PTEN/NLRP3 signaling. **(a)** VSMCs proliferation, **(b)** cell cycle ratio and **(c,d)** migration after CAT-nc and VO-Ophic treatment correspondingly; **(e)**–**(h)** Relative protein expressions of SM22α, α-SMA and OPN; **(i,j)** ROS production level; **(k)**–**(n)** Relative protein expressions of PTEN, NLRP3, P-AKT and AKT. **(o)** Relative content of IL-1β. Data are expressed as mean ± SE (n = 4 independent biological experiments). ^*^ *P < 0.05,* ^**^ *P < 0.01,* ^***^ *P < 0.001,* ^****^ *P < 0.0001*, compared with model; ^*#*^ *P < 0.05,* ^*##*^ *P < 0.01,* ^*###*^ *P < 0.001,* ^*####*^ *P < 0.0001*, compared with control.

To further determine whether the PTEN/NLRP3 signaling pathway is a key target of CAT-nc, subsequent experiments employed the PTEN inhibitor VO-Ohpic at a concentration of 0.1 μM. Co-administration of CAT-nc and VO-Ohpic significantly reversed all inhibitory effects of CAT-nc on vascular smooth muscle cell proliferation and migration (Figures 4a–m). Treatment with VO-Ohpic alone had no effect on normal vascular smooth muscle cells. These results indicate that inhibition of PTEN activity effectively antagonizes the key biological effects of CAT-nc. This suggests that CAT-nc’s regulation of VSMC phenotypic switching depends on PTEN activity. The PTEN/NLRP3 signaling pathway is a key target pathway through which CAT-nc modulates Ang II-induced VSMC phenotypic switching and dysfunction. CAT-nc exerts its anti-proliferative and pro-contractile phenotypic switching effects by activating PTEN and inhibiting NLRP3 inflammasome. CAT-nc significantly reduced the expression level of the AKT protein and decreased the inflammatory expression level of IL-1β (Figures 4n,o).

## 4 Discussion

VascularVascular remodeling is a core pathological basis for various cardiovascular diseases, including hypertension, atherosclerosis, and restenosis, in which phenotypic switching of VSMCs plays a decisive role (Méndez-Barbero et al., 2021). Ang II, a key effector of the renin-angiotensin system, is a central inducer of VSMC phenotypic switching and dysfunction (Xiang et al., 2020; Fang et al., 2025). Network pharmacology and molecular docking analyses predicted a favorable interaction between CAT and PTEN. In this study, CAT-nc significantly counteracted the pro-proliferative and pro-migratory effects of Ang II in a time- and dose-dependent manner, with the most pronounced inhibitory effect observed at 0.3 μg/mL. CAT-nc inhibited cell migration, upregulated the contractile phenotype markers α-SMA and SM22α, and downregulated the synthetic phenotype marker OPN (Ehrlich et al., 2006; Okamoto et al., 2024). These results indicate that the nano-encapsulated catalase delivery system effectively intervenes in key aspects of pathological vascular remodeling at the cellular level. Oxidative stress is a central mechanism driving vascular dysfunction. Ang II stimulation significantly increased intracellular ROS levels in VSMCs, whereas CAT-nc treatment effectively scavenged these ROS. Importantly, co-treatment with the ROS scavenger NAC nearly completely reversed the protective effects of CAT-nc. This robustly confirms that the antioxidant action of CAT-nc is the core initial step in its inhibition of VSMC phenotypic switching.

An interesting observation in this study is the non-monotonic dose-response of CAT-nc, with inhibitory effects on VSMC proliferation evident at 0.1–1 μg/mL but absent at 3 and 10 μg/mL. Although not the main focus, this phenomenon warrants brief discussion. One possible explanation is that excessive ROS scavenging at higher doses may unintentionally disrupt basal redox signaling essential for VSMC homeostasis, as low-level ROS are known to support normal cellular functions (Lyle and Griendling, 2006; Badran et al., 2020)). Alternatively, higher doses may induce mild cellular stress or saturate endocytic uptake pathways, diminishing net efficacy. No overt cytotoxicity was observed at these concentrations, but subtle sub-lethal effects cannot be excluded. Together, these findings suggest an optimal therapeutic window for CAT-nc and highlight the importance of dose optimization in developing antioxidant nanotherapies for vascular diseases (Manshian et al., 2017; Sousa de Almeida et al., 2021). Low to moderate ROS are essential for VSMC functions via redox-sensitive pathways (Taverne et al., 2013; Zhao et al., 2024) CAT-nc alone maintains homeostatic ROS, whereas excess NAC scavenging disrupts this balance (Samuni et al., 2013) Additionally, CAT-nc acts in specific subcellular compartments while NAC acts globally, potentially disturbing compartment-specific ROS signaling. NAC also has non-antioxidant effectsthat may counteract CAT-nc. Hence, NAC reversal likely involves excessive ROS depletion, disrupted compartmentalized signaling, and off-target NAC activities (Aldini et al., 2018).

This study further reveals that the PTEN/NLRP3 signaling pathway serves as a central hub for the downstream protective effects of CAT-nc. PTEN activity is highly dependent on its redox state, as ROS can inactivate PTEN by oxidizing critical cysteine residues at its active site. CAT-nc treatment significantly upregulated PTEN protein expression in VSMCs, accompanied by a decrease in the expression of its downstream effector, the NLRP3 inflammasome. NLRP3 activation plays an important role in vascular inflammation and atherosclerosis progression, directly promoting VSMC transition toward a pro-inflammatory, pro-proliferative phenotype (Chen et al., 2023). Using the specific PTEN inhibitor VO-Ohpic, we demonstrated that inhibition of PTEN activity completely abolished the regulatory effects of CAT-nc on VSMC proliferation, migration, and phenotypic markers, along with an increase in NLRP3 expression levels.

In summary, this study systematically elucidates the molecular mechanism by which CAT-nc counteracts Ang II-induced VSMC proliferation, migration, and phenotypic switching through scavenging ROS, activating PTEN signaling, and subsequently inhibiting NLRP3 signaling. Encapsulating the unstable native catalase within a nano-carrier significantly improved its stability and bioavailability, demonstrating the considerable potential of nanotechnology in the delivery of cardiovascular therapeutics.

This study has certain limitations. All conclusions are based on *in vitro* cellular experiments; the stability, targeting ability, long-term safety, and ultimate therapeutic efficacy of CAT-nc in complex *in vivo* physiological and pathological environments remain to be validated. Future studies should further evaluate the interventional effects of CAT-nc on key pathological processes such as neointimal formation and plaque stability using animal models of atherosclerosis or vascular injury. Nevertheless, this study establishes a solid theoretical foundation for developing novel nano-therapies that target the PTEN/NLRP3 pathway and enhance antioxidant defense, aiming to prevent and treat cardiovascular diseases associated with vascular remodeling.

## 5 Conclusion

This study aimed to investigate the role of CAT-nc in regulating VSMC phenotypic switching and its underlying molecular mechanisms. The results demonstrated that CAT-nc effectively inhibited Ang II induced proliferation and migration of VSMCs, and modulated their transition from a synthetic to a contractile phenotype. Notably, we identified and validated for the first time that this key biological effect of CAT-nc is primarily achieved by reducing reactive oxygen species (ROS) accumulation, thereby activating PTEN and inhibiting NLRP3 inflammasome activity. This finding provides important experimental evidence for a deeper understanding of the role of oxidative stress in vascular remodeling and for the development of novel nano-therapeutic strategies targeting the PTEN/NLRP3 pathway.

## Data Availability

The datasets presented in this study can be found in online repositories. The names of the repository/repositories and accession number(s) can be found in the article/supplementary material.
